# Reduction of Fracture of Superior Maxillary

**Published:** 1890-07

**Authors:** J. C. Walton

**Affiliations:** Howell, Mich.


Dr. J. C. Walton, of Howell, read a paper on "Reduction of Fracture of Superior Maxillary."

### Reduction of Fracture of Superior Maxillary.

BY J. C. WALTON, D.D.S., HOWELL, MICH.

Thursday, July 4, 1889, F. P., a farm laborer, while in the field at work met with an accident, probably the kick of a horse, as circumstances seem to prove. About 11 o'clock the horse made its appearance at the barn free, and friends some minutes later going back to the fields to learn the cause met him staggering toward the house. Free hemorrhage, loss of teeth, and his dazed condition indicated serious injury, and a physician was called.

The knocking out of two centrals, breaking off of right lateral, fracturing of the alveolus about the incisors and the great fracture extending back through the left superior maxilla as shown on the hard palate, nasal bone and malar suture was discovered.

On the following Saturday two physicians and the writer in council decided that no attempt would be made to remove the fractured pieces of alveolus, but a splint was necessary for the broken jaw.

Swollen, distorted features permitted us to see a little of one bloodshot eye. The face was bruised everywhere. The mouth could not be touched without eliciting a groan. Chloroform was administered, the jaws forced apart and held by the physicians with wire hooks while impressions of both jaws were taken with modelling compound. The uncut model shows well the line of fracture through the hard palate, also how the incisor end of the broken piece was up, and the molar end down. The model was cut on the line of fracture, the articulation restored and the pieces fastened in position, after which the splint, as shown, was made of vulcanized rubber. To insure the easy passage of the splint to place the teeth on the restored models were covered with tin foil (No. 60) before packing the rubber.

The splint was placed in position Sunday morning. A piece of binding wire from an anchor screw set in the cuspid at the gum line, and another from the screw in the splint were crossed and twisted with pliers to bring the piece into position in front. The lower jaw was held firmly against the splint by a skull-cap bandage. The piece could not be forced into position at this time. The incisor end pulled into position the following day, but the molar end was never forced fully up to place, probably on account of comminuted bone.

In spite of threatened complications and alarming high temperature the patient recovered, union taking place quickly. No trouble occurred with the fractured alveolus.

Sixteen days after adjustment the splint was taken off in my office, the patient riding in seven miles for this purpose. A few days later the lower third molar was removed and the other molars ground with a corundum wheel to accommodate the molars of the fractured piece.

The root of the broken lateral has since been removed, absorption has gone on as usual with the alveolus, and except a little abscess at the bridge of the nose and hard palate, no discomfort with his jaw reminds him of the time when a horse played football with his head. The patient remained from first to last in the care of his physician.

